# Computational modelling in source space from scalp EEG to inform presurgical evaluation of epilepsy

**DOI:** 10.1016/j.clinph.2019.10.027

**Published:** 2020-01

**Authors:** Marinho A. Lopes, Leandro Junges, Luke Tait, John R. Terry, Eugenio Abela, Mark P. Richardson, Marc Goodfellow

**Affiliations:** a Living Systems Institute, University of Exeter, Exeter, United Kingdom; b Wellcome Trust Centre for Biomedical Modelling and Analysis, University of Exeter, Exeter, United Kingdom; c EPSRC Centre for Predictive Modelling in Healthcare, University of Exeter, Exeter, United Kingdom; d Department of Engineering Mathematics, University of Bristol, Bristol, United Kingdom; e Cardiff University Brain Research Imaging Centre, School of Psychology, Cardiff University, Cardiff, United Kingdom; f Centre for Systems Modelling and Quantitative Biomedicine, University of Birmingham, Edgbaston, United Kingdom; g Institute for Metabolism and Systems Research, University of Birmingham, Edgbaston, United Kingdom; h Institute of Psychiatry, Psychology and Neuroscience, King’s College London, London, United Kingdom

**Keywords:** Epilepsy surgery, Source mapping, Scalp EEG, Neural mass model, Epileptogenic zone, Epilepsy lateralization

## Abstract

**Objective:**

The effectiveness of intracranial electroencephalography (iEEG) to inform epilepsy surgery depends on where iEEG electrodes are implanted. This decision is informed by noninvasive recording modalities such as scalp EEG. Herein we propose a framework to interrogate scalp EEG and determine epilepsy lateralization to aid in electrode implantation.

**Methods:**

We use eLORETA to map source activities from seizure epochs recorded from scalp EEG and consider 15 regions of interest (ROIs). Functional networks are then constructed using the phase-locking value and studied using a mathematical model. By removing different ROIs from the network and simulating their impact on the network’s ability to generate seizures *in silico*, the framework provides predictions of epilepsy lateralization. We consider 15 individuals from the EPILEPSIAE database and study a total of 62 seizures. Results were assessed by taking into account actual intracranial implantations and surgical outcome.

**Results:**

The framework provided potentially useful information regarding epilepsy lateralization in 12 out of the 15 individuals (p=0.02, binomial test).

**Conclusions:**

Our results show promise for the use of this framework to better interrogate scalp EEG to determine epilepsy lateralization.

**Significance:**

The framework may aid clinicians in the decision process to define where to implant electrodes for intracranial monitoring.

## Highlights

• Computational modelling is combined with scalp EEG to assess epilepsy lateralization.
• Our approach proved useful in informing lateralization in 12 out of 15 individuals studied.
• The framework proposed may be used to aid deciding where to implant intracranial electrodes.

## 1 Introduction

According to the World Health Organization, an estimated fifty million people worldwide have epilepsy. Approximately one third do not respond to anti-epilepsy drugs and are therefore potential candidates for epilepsy surgery (Kwan and Brodie, 2000). Surgery aims to resect the epileptogenic zone (EZ) (Rosenow and Lüders, 2001); the brain area that is necessary and sufficient for the generation of seizures. An evaluation to determine the location of this brain area precedes the surgical procedure (Duncan et al., 2016). Several brain imaging modalities may be employed in this evaluation, namely scalp electroencephalography (EEG) and magnetic resonance imaging (MRI) at an initial stage, possibly followed by other multimodal neuroimaging techniques (see Fig. 1 in Duncan et al., 2016). In particular, intracranial EEG (iEEG) is usually used to complement or clarify information obtained from noninvasive modalities (Jayakar et al., 2016). There is a variety of different iEEG techniques (see Table 4 in Jayakar et al., 2016), which should be selected according to the available information extracted from noninvasive data, semiology, and clinical history (Jayakar et al., 2016). One key decision is whether to place electrodes in one brain hemisphere or both. This is frequently not straightforward. For example, up to 68% of unilateral-onset seizures may show bilateral onset on scalp EEG in mTLE (mesial temporal lobe epilepsy), the most common form of epilepsy (Alarcón et al., 2001). Ictal scalp EEG may even suggest false lateralization (Adamolekun et al., 2011). A poor lateralization hypothesis based on noninvasive modalities may lead to an incorrect placement of intracranial electrodes, which in turn may make surgery ill-advised and potentially unsuccessful if performed (Jayakar et al., 2016).

Many computational methods have been proposed in the last two decades to aid clinicians in identifying epilepsy lateralization using different noninvasive recording modalities, such as scalp EEG (Caparos et al., 2006, Verhoeven et al., 2018), MRI (Keihaninejad et al., 2012, Pustina et al., 2015), and MEG (Wu et al., 2018). Most of these methods aimed to build classifiers using data-driven approaches. For example, Cantor-Rivera et al. (2015) used support vector machines to build a classifier based on diffusion tensor imaging to identify people with TLE. Verhoeven et al. (2018) used functional networks estimated in different frequency bands to build a classification system based on Random Forests classifiers. Indeed, machine learning is an attractive tool to build data-driven classifiers (Jordan and Mitchell, 2015). Although such data-driven methods may in some cases achieve high classification power, they lack a description of the fundamental mechanisms underpinning the phenomena under consideration. They also require sufficiently large datasets, which are often not available. Furthermore, machine learning usually relies on manual labelling of training data, which may be error-prone and time consuming. In the case of epilepsy lateralization, a data-driven approach is unable to describe the mechanisms that may cause the generation of seizures in one hemisphere, making it hard to interpret its predictions together with other clinical information.

In contrast, recent studies have used mathematical models of epilepsy to better interrogate iEEG data and make predictions for epilepsy surgery (Goodfellow et al., 2016, Sinha et al., 2016, Jirsa et al., 2017). In these studies, iEEG was either used to construct functional brain networks (Goodfellow et al., 2016, Sinha et al., 2016), or to validate model parameters (Jirsa et al. 2017). Computational simulations then allowed to make predictions of which brain regions were more likely to be the EZ. Herein we sought to explore whether such methodology when applied to scalp EEG may aid in determining epilepsy lateralization and may be used to inform intracranial electrode implantation. We used 15 individuals from EPILEPSIAE (a European epilepsy database comprising long-term continuous EEG data) (Ihle et al., 2012) and studied a total of 62 seizures. All patients had iEEG, received surgery, and their postsurgical outcome was known. We used exact low-resolution brain electromagnetic tomography (eLORETA) to map source activities from seizure epochs (Pascual-Marqui, 2007, Pascual-Marqui, 2009), and mapped them into a predefined list of 15 regions of interest (ROIs) that were selected according to their established importance across epilepsy syndromes. We then constructed functional networks using the phase-locking value (Tass et al., 1998, Lachaux et al., 1999, Mormann et al., 2000). Finally, the networks were studied using a canonical model of ictogenicity (Lopes et al., 2017) and lateralization was inferred based on the concept of node ictogenicity (Goodfellow et al., 2016, Lopes et al., 2017). This measure assesses the importance of different brain regions in the ability of the network to generate seizures. Our results showed that our scalp EEG based predictions were more likely to be concordant with the performed surgery when the individual had a positive postsurgical outcome and were more often discordant or inconclusive when the individual had a poor outcome.

## 2 Methods

### 2.1 Data

We studied 15 individuals from EPILEPSIAE (Ihle et al., 2012). We used three criteria to choose these individuals: (i) had both intracranial and scalp EEG recordings; (ii) received surgery; and (iii) had at least 12 months follow-up. We used these criteria so that we could compare predictions from scalp EEG with the placement of implanted electrodes and use postsurgical outcome as a validation for whether our predictions could have added value in presurgical evaluation. Each case had a different electrode implantation scheme, which included grid, strip and depth electrodes. 5 individuals had a bilateral electrode implantation. Scalp EEG was recorded using the 10–20 system for electrode placement. The standard 19 channels were considered (T1, T2, FP1, F7, FP2, F3, F4, C4, P3, P4, O1, O2, T3, T4, T5, T6, Fz, Cz, C3, F8, and Pz). 10 individuals achieved a positive postsurgical outcome (Engel class Ia and Ib), and 5 had a poor outcome (Engel class IIa and IIIa). Table 1 contains a summary of the clinical details relevant for this study, namely the foci identified from intracranial EEG and surgery localization.

**Table 1:** Clinical characteristics of the individuals considered in this study. The first column identifies the patients’ ID, the second indicates their gender (F = female, M = male), and the third their age in years. The electrode implantation column specifies whether intracranial electrodes were implanted either in the right or in the left hemispheres or both (bilateral). Focus in intracranial EEG indicates the region or regions that were identified during monitoring (the numbers sort the foci by importance, with higher numbers denoting regions of lower relevance). Surgery localisation defines the brain region targeted by the performed surgery (established from an MRI after surgery). The outcome column describes the postsurgical outcome achieved by each individual according to the Engel classification measured at least 12 months after surgery. The last column on the right indicates the number of seizures (# of sz.) used in this study that follow the criteria described in the text.

Patient ID	Gender	Age	Electrode implantation	focus in intracranial EEG	Surgery localization	Outcome	# of sz.
FR 115	M	34	right	temporal mesial right	temporal right	Ia	5
FR 253	F	37	bilateral	(1) temporal mesial left; (2) temporal mesial right	temporal right	Ia	4
FR 384	F	50	right	frontal right	frontal right	Ia	4
FR 442	M	21	right	(1) temporal lateral right; (2) temporal mesial right	temporal right	Ia	5
FR 548	M	17	bilateral	(1) temporal mesial left; (2) temporal lateral left	temporal left	Ia	4
FR 590	M	18	bilateral	(1) temporal basal left; (2) temporal lateral left; (3) temporal basal right	temporal left	Ia	1
FR 916	M	23	left	temporal mesial left	temporal left	Ib	5
FR 958	F	14	left	(1) temporal left; (2) temporal lateral left	none (no MRI)	Ia	1
FR 1096	F	32	bilateral	temporal mesial left	temporal left	Ia	5
FR 1125	F	11	right	temporal mesial right	temporal right	Ia	4
FR 273	F	3	left	(1) temporal mesial left; (2) temporal lateral left	temporal left	IIIa	5
FR 583	F	22	left	temporal lateral left	temporal left	IIa	5
FR 818	F	27	left	temporal left	temporal left	IIIa	4
FR 970	M	15	right	temporal basal right	temporal right	IIa	5
FR 1073	F	47	bilateral	(1) temporal mesial right; (2) temporal lateral right	temporal right	IIIa	5

For each individual, we selected from the available scalp EEG data up to 5 seizures according to the following criteria: a seizure had to be at least 1 h apart from other seizures or subclinical events and be at least 16 seconds long. The first criterion aimed at increasing the chance of analyzing independent and informative seizures. For example, two succeeding seizures may be less informative, as the second may be provoked by the first, and therefore predictions based on the two seizures may not be independent. The second criterion was used to make sure we had enough data samples per seizure for subsequent analysis. In individuals with more than 5 seizures, we selected the first 5 that obeyed the criteria. We considered 62 seizures in total, with an average seizure duration of 102.9 ± 52.5 s. Table 1 indicates the number of seizures considered per individual.

EEG data was recorded at sampling rates of 256, 512, and 1024 Hz. For consistency, all data were down-sampled to 256 Hz. Furthermore, we applied a broadband (1–25 Hz) band-pass filter (fourth-order Butterworth filter with forward and backward filtering to minimize phase distortions). This frequency band contains the traditional clinical frequency bands (delta, theta, alpha, and most of beta (Buzsaki, 2006)), while avoiding high frequencies which may be corrupted with muscle electrical activity (Whitham et al., 2007).

### 2.2 Source mapping

For each seizure considered, cortical source mapping was performed using the Fieldtrip toolbox (Oostenveld et al., 2011; http://www.ru.nl/neuroimaging/fieldtrip). The Montreal Neurological Institute ‘ICBM152_2016’ average MRI (Mazziotta et al., 2001) implemented in the Brainstorm software (Tadel et al. 2011) was used to develop a 3-layer boundary element method head model (Fuchs et al., 2002) and a 8004 voxel cortical source space limited to the grey matter cortical surface. Use of template models has previously been demonstrated to perform well compared to individual models derived from MRI (Fuchs et al., 2002). Dipoles were oriented normal to the surface of the cortical sheet (Hassan et al., 2014).

We used exact low-resolution brain electromagnetic tomography (eLORETA) to solve the inverse problem and reconstruct source activity at each of the 8004 source points (Pascual-Marqui, 2007, Pascual-Marqui, 2009). eLORETA is a linear, regularized, weighted minimum norm inverse solution with theoretically exact zero error localization even in the presence of structured biological or measurement noise (Pascual-Marqui, 2007). It has been shown to be appropriate for the study of whole brain phase synchronization (Pascual-Marqui et al., 2011, Finger et al., 2016), and the LORETA family of solutions has been validated against numerous imaging modalities (Dierks et al., 2000, Vitacco et al., 2002, Mulert et al., 2004, Pizzagalli et al., 2004, Zumsteg et al., 2005, Zumsteg et al., 2006, Olbrich et al., 2009) and simulations (Pascual-Marqui et al., 2011, Finger et al., 2016).

### 2.3 Regions of interest

The human EEG captures signals that arise from postsynaptic potentials generated in regions of the cerebral cortex (Olejniczak, 2006, Cohen, 2017). These regions need to be sufficiently large to produce measurable signals (6–30 cm^2^) (Rose and Ebersole, 2009). Due to volume conduction, EEG scalp potentials reflect a time-dependent sum of activity from many cortical regions. Finding individual regions from ongoing EEG is therefore ill-posed, and neuroanatomical assumptions are needed to obtain plausible solutions (Michel et al., 2004). Here, we selected a set of neuroanatomical ROIs for EEG source mapping that are relevant for epilepsy. Although epilepsy can arise from multiple different neuroanatomical regions, there is a set of core areas that appear to be affected across epilepsy syndromes (Richardson, 2012, O’Muircheartaigh and Richardson, 2012, Besson et al., 2017). These regions can be mapped onto three intrinsic “attentional networks”: the default mode network, the salience network, and the frontoparietal control network (Besson et al., 2017, Pittau et al., 2012, de Campos et al., 2016). Table 2 specifies these networks, the brain areas involved, and the respective regions of interest (ROIs) identified in the Desikan-Killiany atlas (Desikan et al., 2006). Note that due to the intrinsically low spatial resolution of EEG, we fused some of the midline ROIs (see the ROIs identified with an asterisk in Table 2). We consider 15 ROIs in total.

**Table 2:** Regions of interest (ROIs) selected for source mapping. The left column presents the brain networks considered, the middle column the brain areas involved in each network, and the right column the regions that were chosen from the Desikan-Killiany atlas as representative of these areas for our analysis. The selected ROIs represent a compromise between mapping regions from the three networks considered and the number of EEG channels used in this study. Furthermore, deep brain regions were not considered since these are unlikely to be recorded with EEG. Note that ROIs identified with an * comprised both left and right regions, meaning that we merged them (these were regions close to the brain’s midline). Note that the rostral middle frontal region appears twice on the right column because it belongs to both the default mode network and frontoparietal control network.

Network	Brain area	Chosen ROI in the Desikan-Killiany atlas
Default mode network	Dorsal medial prefrontal cortex	Medial orbito frontal*
	Rostral anterior cingulate	Rostral anterior cingulate*
	Lateral frontal cortex (superior frontal cortex and inferior frontal gyrus)	Rostral middle frontal*
	Medial parietal cortex (posterior cingulate and retrosplenial cortex)	Precuneus*
	Medial temporal lobe (hippocampus and parahippocampal cortices)	Parahippocampal left
		Parahippocampal right
	Lateral parietal cortex (angular gyrus and posterior supramarginal gyrus/TPJ)	Supramarginal left
		Supramarginal right
	Lateral temporal cortex (including temporal poles)	Superior temporal left
		Superior temporal right

Salience network	Dorsal anterior cingulate cortex	Caudal anterior cingulate*
	Anterior insulae	Insula left
		Insula right

Frontoparietal control network	Dorsolateral prefrontal cortex	Rostral middle frontal*
	Posterior parietal cortex	Superior parietal left
		Superior parietal right

Parcellation was performed by taking the first principal component of all source points within a given ROI in order to construct a single time series for that ROI (Hassan and Wendling, 2018, Tait et al., 2019). For eLORETA solutions, which constrain spatial smoothness and are low resolution, the activity of local voxels is highly correlated. The time course of the first principal component of all voxels in the ROI is a single time series whose value at each time point is minimally different to the activity of all voxels, i.e. it accounts for a maximal spatial variance.

### 2.4 Functional network

Following the procedure above, for each considered seizure epoch we obtained 15 time series describing the seizure dynamics within the selected ROIs. We then divided the time series in consecutive nonoverlapping segments of 16 seconds (4096 data samples, a choice that is a compromise between needing a sufficient number of samples for further analysis, being a power of 2 for computational efficiency, and signal stationarity (Rummel et al., 2015)). Functional networks were constructed from each segment (15 ROIs × 4096 data samples) using the Phase Locking Value (PLV) (Tass et al., 1998, Lachaux et al., 1999, Mormann et al., 2000, Le Van Quyen et al., 2001, Aydore et al., 2013). ROIs were considered as network nodes, and weight connections between pairs of ROIs i and j were calculated as

$$\mathrm{PL}V_{\mathrm{ij}}=\frac{1}{N_{s}}\left|\sum_{k=1}^{N_{s}}e^{i\Delta \phi_{\mathrm{ij}}\left(t_{k}\right)}\right|$$

where $N_{s}$ is the number of samples ($N_{s}=4096$), and $\Delta \phi_{\mathrm{ij}}\left(t_{k}\right)$ is the instantaneous phase difference between the time series from ROI i and j at time $t_{k}$. These phase differences were computed using the Hilbert transform. We then excluded spurious connections by comparing the PLV values to other PLV values computed from surrogate time series. We generated 99 surrogates from the signals of the ROIs using the iterative amplitude-adjusted Fourier transform (IAAFT) with 10 iterations (Schreiber and Schmitz, 1996, Schreiber and Schmitz, 2000) and computed 99 PLV values of every pair of ROIs. PLV values from the original ROIs that did not exceed the 95% significance level compared to the corresponding PLV values from the surrogates were rejected. Thus, the functional networks considered in this study are weighted and correspond to the matrices of statistically significant PLV values.

### 2.5 Mathematical model

To study the importance of different ROIs to the network’s ability to generate seizures, we placed a canonical mathematical model of ictogenicity at each network node (Goodfellow et al., 2016, Lopes et al., 2017, Lopes et al., 2018, Lopes et al., 2019). Within the model, nodes’ activity was described by a phase oscillator $\theta_{i}$. Two states were defined: ‘resting state’ when the oscillator fluctuated close to a fixed stable phase $\theta^{\left(s\right)}$ and a ‘seizure state’ corresponding to a rotating phase. Oscillators’ time dependence was described by the theta model (Lopes et al., 2017, Lopes et al., 2018, Lopes et al., 2019):

$$\dot{\theta_{i}}=\left(1-\cos\theta_{i}\right)+\left(1+\cos\theta_{i}\right)I_{i}\left(t\right)$$

where $I_{i}\left(t\right)$ is the input current received by node i at time t. This current comprised noise and the interaction with other oscillators in the network:

$$I_{i}\left(t\right)=I_{0}+\xi^{\left(i\right)}\left(t\right)+\frac{K}{N}\sum_{i\neq j}a_{\mathrm{ji}}\left[1-\cos\left(\theta_{j}-\theta^{\left(s\right)}\right)\right]$$

where $I_{0}+\xi^{\left(i\right)}\left(t\right)$ represents Gaussian noise, K is a global scaling factor of the network’s interaction, N is the number of nodes (N=15), and $a_{\mathrm{ji}}$ is the j,i^th^ entry of the weighted adjacency matrix representing the functional network. The noise aims to account for signals coming from remote brain regions outside of the functional network under consideration. This model describes a saddle-node on invariant circle (SNIC) bifurcation at $I_{i}=0$, which separates the resting state ($I_{i}<0$) and the seizure state ($I_{i}>0$). This simple model has been shown to approximate the interaction between neural masses (Lopes et al., 2017). Parameters were chosen according to previous studies (Lopes et al., 2017, Lopes et al., 2018, Lopes et al., 2019): $I_{0}=-1.2$ and noise standard deviation σ=0.6. The global scaling factor K was used as a free parameter (see Section 2.6).

### 2.6 Node Ictogenicity

To measure the relative importance of each ROI to the network’s ability to generate seizures, we computed the *Node Ictogenicity* (NI) (Goodfellow et al., 2016, Lopes et al., 2017, Lopes et al., 2019). The NI concept was first introduced in (Goodfellow et al., 2016), and it quantifies the effect of removing nodes on the networks ability to generate seizures. In turn, the networks ability to generate seizures can be measured using the concept of *Brain Network Ictogenicity* (BNI), which is the fraction of time that the network spends in the seizure state (Petkov et al., 2014):

$$\mathrm{BNI}=\frac{1}{N}\sum_{i}\frac{t_{\mathrm{sz}}^{\left(i\right)}}{T}$$

where $t_{\mathrm{sz}}^{\left(i\right)}$ is the time that node i spends in the oscillatory state during a total simulation time T (we used $T=4\times 10^{6}$, as in (Lopes et al., 2019); see Lopes et al. (2017) for more details on the calculation of $t_{\mathrm{sz}}^{\left(i\right)}$). NI was then calculated as

$$NI^{\left(i\right)}=\frac{\mathrm{BN}I_{\mathrm{pre}}-BNI_{\mathrm{post}}^{\left(i\right)}}{\mathrm{BN}I_{\mathrm{pre}}}$$

where $\mathrm{BN}I_{\mathrm{pre}}$ is BNI prior to node removal, and $\mathrm{BN}I_{\mathrm{post}}^{\left(i\right)}$ is BNI after the removal of node i. As in our previous works, we selected the parameter K such that $\mathrm{BN}I_{\mathrm{pre}}=0.5$ (Goodfellow et al., 2016, Lopes et al., 2017, Lopes et al., 2019). $\mathrm{BN}I_{\mathrm{post}}^{\left(i\right)}$ is typically equal or smaller than $\mathrm{BN}I_{\mathrm{pre}}$, depending on whether the node i contributes to seizure generation. If the removal of node i stops the network from generating seizures ($\mathrm{BN}I_{\mathrm{post}}^{\left(i\right)}=0$), then $NI^{\left(i\right)}=1$, whereas if it plays no role in seizure generation ($\mathrm{BN}I_{\mathrm{post}}^{\left(i\right)}=BNI_{\mathrm{pre}}$), then $NI^{\left(i\right)}=0$. In this study we were interested in identifying the ROIs with the highest NI.

### 2.7 Lateralization

**Fig. 1:**
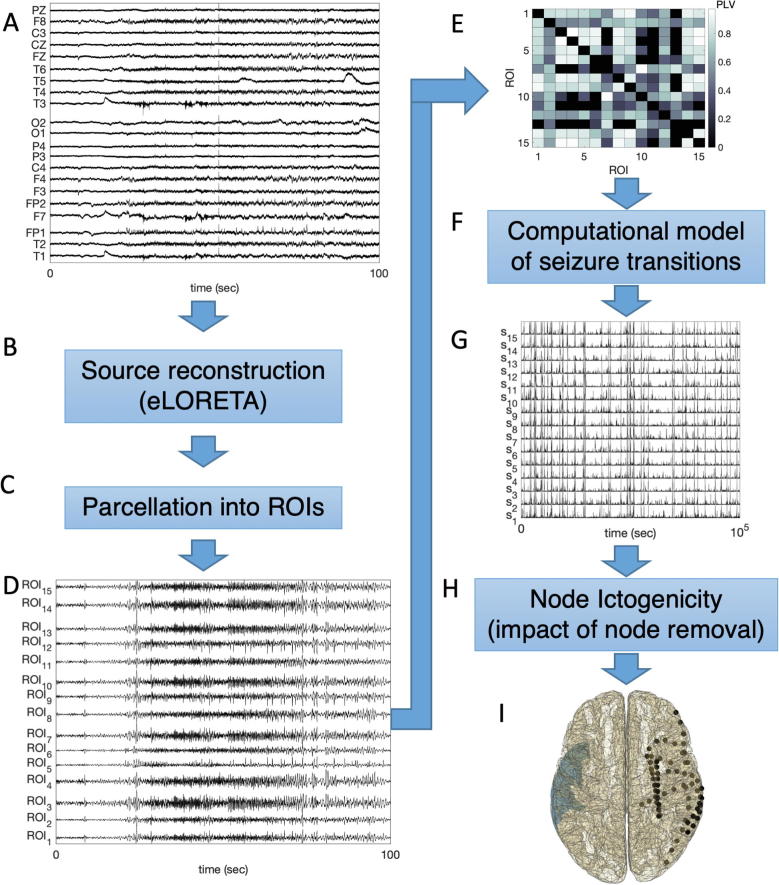
Scheme of the data analysis procedure. (A) 19-channel scalp EEG recordings containing seizures are considered. (B) Cortical source mapping is performed using eLORETA. (C) 15 ROIs are studied by taking the first principal component from all sources within the regions. (D) Example time series of the ROIs reconstructed from the signals displayed in (A). (E) Functional networks are inferred from the signals of the ROIs using the PLV. (F) A computational model of ictogenicity (the theta model) is employed to simulate dynamics on the networks. (G) Example times series generated using the theta model on the network (E). (H) The NI is computed by measuring the impact of removing nodes on the network’s ability to generate seizures *in silico*. (I) The ROI with the highest NI is identified (colored blue) and the prediction is compared with intracranial electrode implantation (black dots), performed surgery and postsurgical outcome (metadata not represented here). The comparison consists of observing whether the ROI with highest NI is in the same hemisphere where surgery was performed, and whether it is concordant with intracranial electrode placement. The aim is to observe whether this framework could have added value to the clinical decision-making process of defining where to implant intracranial electrodes to map the epileptogenic zone. (For interpretation of the references to colour in this figure legend, the reader is referred to the web version of this article.)

To extract a prediction based on our framework of which brain hemisphere is more likely to contain the epileptogenic zone, we identified the ROIs with highest NI. The maximum NI resected as computed from intracranial EEG functional networks has been shown to be able to predict postsurgical outcome (see Fig. 4b in Goodfellow et al., 2016). Given that we obtained functional networks for each 16-second segment of each seizure, we first found the ROIs that consistently presented higher NI within single seizures. Furthermore, since we analyzed multiple seizures per individual, we then gathered together one predicted ROI per seizure. Finally, a consensus analysis was performed by which the most frequent ROI across seizures was identified. In cases where two or more ROI located in both hemispheres were identified as equally frequent, we defined the prediction as inconclusive. These ROIs are then compared to the placement of electrode implantation, the surgery localization, and patient postsurgical outcome (see Table 1). Fig. 1 summarizes the key steps of our methods.

## 3 Results

The NI framework described in the Methods has been shown to be able to extract relevant information from iEEG in the context of epilepsy surgery (Goodfellow et al., 2016, Lopes et al., 2017, Lopes et al., 2018). Here we aimed to explore whether the same framework could yield useful information for presurgical evaluation when applied to source mapped data from scalp EEG using relevant ROIs. As summarized in Fig. 1, our methods consisted in (i) mapping cortical sources using eLORETA applied to scalp EEG, (ii) parcellating the sources into ROIs, (iii) inferring functional networks, and (iv) computing NI to determine lateralization. Note, however, that in this preliminary study we do not attempt to localize the specific brain region responsible for seizure generation. On one hand we do not expect source mapping based on 19-channel EEG to have sufficient spatial resolution for this purpose, and on the other hand the specific region targeted by surgery is not indicated in the EPILEPSIAE database.

**Fig. 2:**
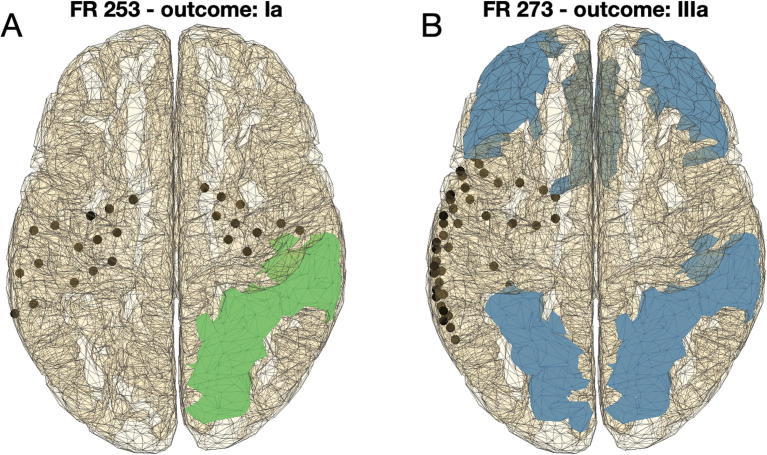
Two exemplar applications of the framework to individuals with good and bad postsurgical outcome. (A) Patient FR 253 had a bilateral intracranial electrode implantation (see black dots), and the performed surgery targeted a region in the right hemisphere (not represented). The patient achieved a good postsurgical outcome (Engel Ia). Four seizures recorded from scalp EEG were analyzed using our framework and two candidate regions for resection were identified in the right hemisphere (superior parietal and supramarginal; regions highlighted in green), concordant with the hemisphere where surgery was performed. (B) Patient FR 273 had intracranial electrodes implanted in the left hemisphere, and the performed surgery targeted a region in the left hemisphere. The postsurgical outcome was poor (Engel IIIa). In this case we studied five seizures and each of them identified a different possible candidate region for resection (regions highlighted in blue). Such inconclusive result from scalp EEG would support a bilateral electrode implantation. (For interpretation of the references to colour in this figure legend, the reader is referred to the web version of this article.)

Fig. 2 shows the ROIs identified in two individuals using our framework. Individual FR 253 had a bilateral intracranial electrode implantation, received surgery on the right hemisphere and the individual achieved seizure freedom (Engel class Ia). Application of the NI framework identified the regions in the right hemisphere (superior parietal and supramarginal regions) in line with the performed surgery. In this case, our methods could suggest that a bilateral electrode implantation had been unnecessary, and instead an implantation on the right hemisphere could have sufficed. In contrast, individual FR 273 had intracranial electrodes implanted on the left hemisphere, surgery targeted the left hemisphere, and the individual continued to experience seizures after the surgery (Engel class IIIa). In this case, the NI framework applied to scalp EEG was unable to lateralize the epileptogenic zone, i.e. it identified regions in both hemispheres. This result might indicate a bilateral implantation of intracranial electrodes, which could help determine whether a single epileptogenic zone was located in the left or right hemisphere, or whether there were multiple epileptogenic zones.

**Fig. 3:**
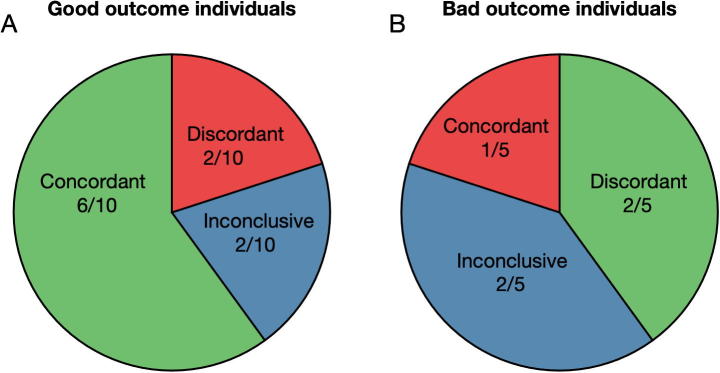
Summary of individual comparison of performed surgeries and framework predictions based on scalp EEG stratified by postsurgical outcome: (A) good postsurgical outcome individuals and (B) bad postsurgical outcome individuals. Concordant (discordant) indicates the fraction of individuals for which the framework prediction was concordant (discordant) with the performed surgery. Inconclusive represents the cases in which the framework was uncapable of identifying one hemisphere as more likely to contain the epileptogenic zone. Note that we colored the cases where the framework could be useful with green (concordant in good outcome individuals and discordant in bad outcome individuals); with red where predictions may be inadequate; and with blue where the predictions were inconclusive (and therefore potentially useful, particularly in the bad outcome cases). (For interpretation of the references to colour in this figure legend, the reader is referred to the web version of this article.)

Similar interpretations to those derived from Fig. 2 were applied individually to the 15 patients considered in this study (see the Supplementary Fig. 1 and Supplementary Table 1). Our results are summarized in the two columns on the right of Table 3. Predictions were classified as either concordant if in agreement with the performed surgery, discordant if not in agreement with the performed surgery, and inconclusive if unable to lateralize the responsible area for the seizures. The value of a prediction being concordant, discordant or inconclusive was considered to depend on whether the performed surgery achieved a good postsurgical outcome. We therefore summed the different types of prediction stratified by postsurgical outcome. Fig. 3 shows that in good outcome individuals, 6 of our predictions were concordant with the performed surgeries, 2 were discordant and 2 were inconclusive. In contrast, in bad outcome individuals the predictions were only concordant in one individual and inconclusive and discordant in the remaining individuals. In general, the framework could provide potentially useful information for all individuals except the 2 discordant good outcome individuals and the one concordant bad outcome individual (red slices in the figure).

**Table 3:** Clinical characteristics of the individuals considered in this study and epilepsy lateralization predicted. As in Table 1, the first column identifies the patients’ ID. The outcome column describes their postsurgical outcome (we consider Engel Ia and Ib good outcome, and IIa and IIIa bad outcome). The electrode implantation column specifies whether intracranial electrodes were implanted either in the right or in the left hemispheres or both (bilateral). Focus in intracranial EEG indicates the region or regions that were identified during monitoring (the numbers sort the foci by importance, with higher numbers denoting regions of lower relevance). Surgery localisation defines the brain region targeted by the performed surgery (established from an MRI after surgery). The next column to the right indicates the number of seizures (# of sz.) used in this study that follow the criteria described in the text. The column prediction presents the lateralization as predicted from our framework. Finally, the last column clarifies whether the predictions are concordant (C), discordant (D) or inconclusive (I) compared to the surgery localization.

Patient ID	Outcome	Electrode implantation	focus in intracranial EEG	Surgery localization	# of sz.	Prediction	CDI
FR 115	Ia	right	temporal mesial right	temporal right	5	right	C
FR 253	Ia	bilateral	(1) temporal mesial left; (2) temporal mesial right	temporal right	4	right	C
FR 384	Ia	right	frontal right	frontal right	4	right	C
FR 442	Ia	right	(1) temporal lateral right; (2) temporal mesial right	temporal right	5	left	D
FR 548	Ia	bilateral	(1) temporal mesial left; (2) temporal lateral left	temporal left	4	left	C
FR 590	Ia	bilateral	(1) temporal basal left; (2) temporal lateral left; (3) temporal basal right	temporal left	1	left	C
FR 916	Ib	left	temporal mesial left	temporal left	5	left	C
FR 958	Ia	left	(1) temporal left; (2) temporal lateral left	none (no MRI)	1	inconclusive	I
FR 1096	Ia	bilateral	temporal mesial left	temporal left	5	right	D
FR 1125	Ia	right	temporal mesial right	temporal right	4	inconclusive	I
FR 273	IIIa	left	(1) temporal mesial left; (2) temporal lateral left	temporal left	5	right	D
FR 583	IIa	left	temporal lateral left	temporal left	5	left	C
FR 818	IIIa	left	temporal left	temporal left	4	inconclusive	I
FR 970	IIa	right	temporal basal right	temporal right	5	inconclusive	I
FR 1073	IIIa	bilateral	(1) temporal mesial right; (2) temporal lateral right	temporal right	5	left	D

We tested the hypothesis of whether our results could be obtained by chance, namely whether the fraction of potentially useful predictions (12 out of 15) could be achieved by a random predictor and found a p-value of 0.02 (binomial test). Thus, our results are statistically significant at the significance level of 0.05.

## 4 Discussion

In this study we posed the question as to whether a previously proposed framework to interrogate iEEG to inform epilepsy surgery could be extended to assess scalp EEG with the aim of improving its value in the presurgical decision-making process, particularly in inferring epilepsy lateralization. The framework to explore iEEG data (Goodfellow et al., 2016) consisted in building a functional network from the data and examine it by placing a mathematical model of epilepsy into the network. Computer simulations of the model then enabled to study the effect of different node removals from the network on the overall propensity of the network to generate seizure dynamics *in silico*. The framework was validated in a cohort of 16 patients that underwent epilepsy surgery, and it showed that patients who had a good postsurgical outcome received surgeries that aligned better with optimal surgeries as predicted by the framework than patients who did not. Similarly, here we applied the framework to source mapped data from scalp EEG of 15 individuals who received epilepsy surgery (EPILEPSIAE database). Source activity was inferred using eLORETA, and sources were parcellated into 15 ROIs belonging to the default mode network, the salience network, and the frontoparietal control network (see Table 2). These networks were chosen as they have been found to play a role across different epilepsy syndromes (Richardson, 2012, O’Muircheartaigh and Richardson, 2012, Besson et al., 2017). For each individual, we studied up to 5 different seizures (see Table 1) and extracted conclusions based on a consensus analysis of the most ictogenic ROIs identified from each seizure. We divided the patients into two groups: good postsurgical outcome (Engel class Ia and Ib) and poor postsurgical outcome (Engel class IIa and IIIa). In good postsurgical outcome cases, we expected that most of our predictions should agree with the location of resection in the performed surgery. Indeed, in 6 out of 10 individuals who had good outcome the framework identified ROIs with the highest ictogenicity in the operated brain hemisphere. In the other 4 individuals in this group the framework was either inconclusive (2/10) or discordant (2/10) compared to the actual performed surgery. Note that inconclusive cases could potentially become conclusive by adding more seizure epochs to the analysis. If such ambiguity would remain, this could be interpreted as advising the use of bilateral iEEG, which could in turn disambiguate these results from noninvasive EEG. In contrast, in the poor outcome group, only 1 out of 5 individuals received surgery with resection location concordant with the lateralization predicted by our framework. Given that for this group we would expect that the performed surgeries would disagree with the framework predictions, we have to acknowledge a number of further confounding factors. First, even if lateralization was correctly identified during presurgical evaluation, this does not guarantee that the surgery should be successful, as it may have not targeted the EZ, or may not have removed a sufficient portion of it. Also, overlap between the EZ and eloquent cortex could have limited the extent of the surgical resection. For the other 4 individuals with bad outcome, the framework was inconclusive in 2 and discordant with the performed surgery in the other 2. As above, the inconclusive cases could potentially be disambiguated by considering more seizure epochs or could indicate the use of bilateral iEEG monitoring. Interestingly, in all 4 cases where our framework was inconclusive (in both good and bad outcome cases), all these individuals did not have bilateral implanted iEEG, but at least in the 2 bad outcome cases could have potentially benefited from it. Bilateral electrode implantation was used in 5 individuals (see Table 1), 4 with good postsurgical outcome and 1 with bad postsurgical outcome. The framework was concordant with 3 of the surgeries performed in the good postsurgical outcome, suggesting that the bilateral implantation could have been avoided in these cases. In the bad outcome case with bilateral iEEG (FR 1073), the framework was discordant with the performed surgery, suggesting that a more careful mapping of the left hemisphere could have been valuable.

A number of data-driven approaches have been explored to build classifiers of epilepsy lateralization from scalp EEG (Caparos et al., 2006, Verhoeven et al., 2018). In Caparos et al. (2006), the authors observed that nonlinear correlation coefficients were higher on the side where seizures started, and this could be used as a marker of seizure lateralization. More recently, Verhoeven et al. (2018) produced the first automatic tool for diagnosis and lateralization of temporal lobe epilepsy using scalp EEG and machine learning. As we commented in the Introduction, such methods may achieve good classification, but their results may be difficult to interpret at an individual basis and together with other clinical information given that their output is usually binary. A more mechanistic description such as the one proposed here opens avenues to integrate information from different data modalities and may be more helpful in the decision-making process during presurgical evaluation.

The results of our study are potentially confounded by a number of factors. We acknowledge that the dataset used in this work is small. Whilst we aim for person-specific predictions, valid for use in pre-surgical planning, larger data sets would help us to more accurately quantify the percentage of people for whom the framework is expected to be useful. As more data becomes publicly available, future studies will facilitate this. Furthermore, as more data is added into the analysis, more tailored predictions may be possible, by taking into account possible confounding factors such as epilepsy syndrome and epilepsy duration. More data will also provide the opportunity to optimize the preliminary methodology presented here. For example, here we examined scalp EEG in a broad frequency band between 1 and 25 Hz. Results could potentially be improved using other frequency bands (Schmidt et al., 2014). More seizure epochs per individual would also be useful, as it would enable a more robust analysis. This would enable to examine the variability in lateralization. Such analysis is crucial to determine the value of any biomarker, as it has been recently exemplified in the case of HFOs (Gliske et al., 2018). Future studies should also consider using other data segments other than seizures. For example, it may be tested whether our framework could be applied to functional networks inferred from interictal epileptiform discharges (IEDs). Coito et al. (2016) have inferred functional connectivity from IEDs and showed that people with temporal lobe epilepsy have reduced connectivity in the default mode network compared to healthy controls. The two methodologies could be merged, and results could be compared using IEDs and seizure epochs. Furthermore, here we decided to study 15 ROIs from the default mode network, the salience network, and the frontoparietal control network. A bias towards temporal epilepsies cannot be excluded, but these networks may be a useful first approach. Future studies may explore other networks and different numbers of ROIs. It would also be worth exploring how predictions change according to the number of electrodes considered in scalp EEG. It has been shown that higher electrode densities enable a more accurate source localization (Lu et al., 2012). This would allow us to consider and compare denser ROI parcellations, and potentially better resolve midline parcellations which in the current approach comprise one third of all ROIs considered, but do not provide information on epilepsy lateralization. Finally, in this study we used a template head model for source mapping. Although it has been shown that template models perform well compared to individual models constructed from MRI (Fuchs et al., 2002), the use of personalized head models may further optimize our framework.

## 5 Conclusions

In summary, our results show promise that a framework based on functional networks inferred from scalp EEG and their analysis by the use of computational models of ictogenicity may be informative in the presurgical evaluation process, particularly for deciding the placement of intracranial EEG electrodes. It may also be useful in resource-poor countries, where access to expensive neuroimaging techniques may be limited (Radhakrishnan, K., 2009), and therefore there is a need to make a better use of scalp EEG.

## Declaration of Competing Interest

JT is co-founder and Director of Neuronostics.
