# Supplementary material for: Computational modelling in source space from scalp EEG to inform presurgical evaluation of epilepsy
Source: Clin Neurophysiol. 2020 Jan;131(1):225–34. doi: 10.1016/j.clinph.2019.10.027 (PMC6941468; doi:10.1016/j.clinph.2019.10.027)
Supplement: Supplementary data 1 [file mmc1.docx]

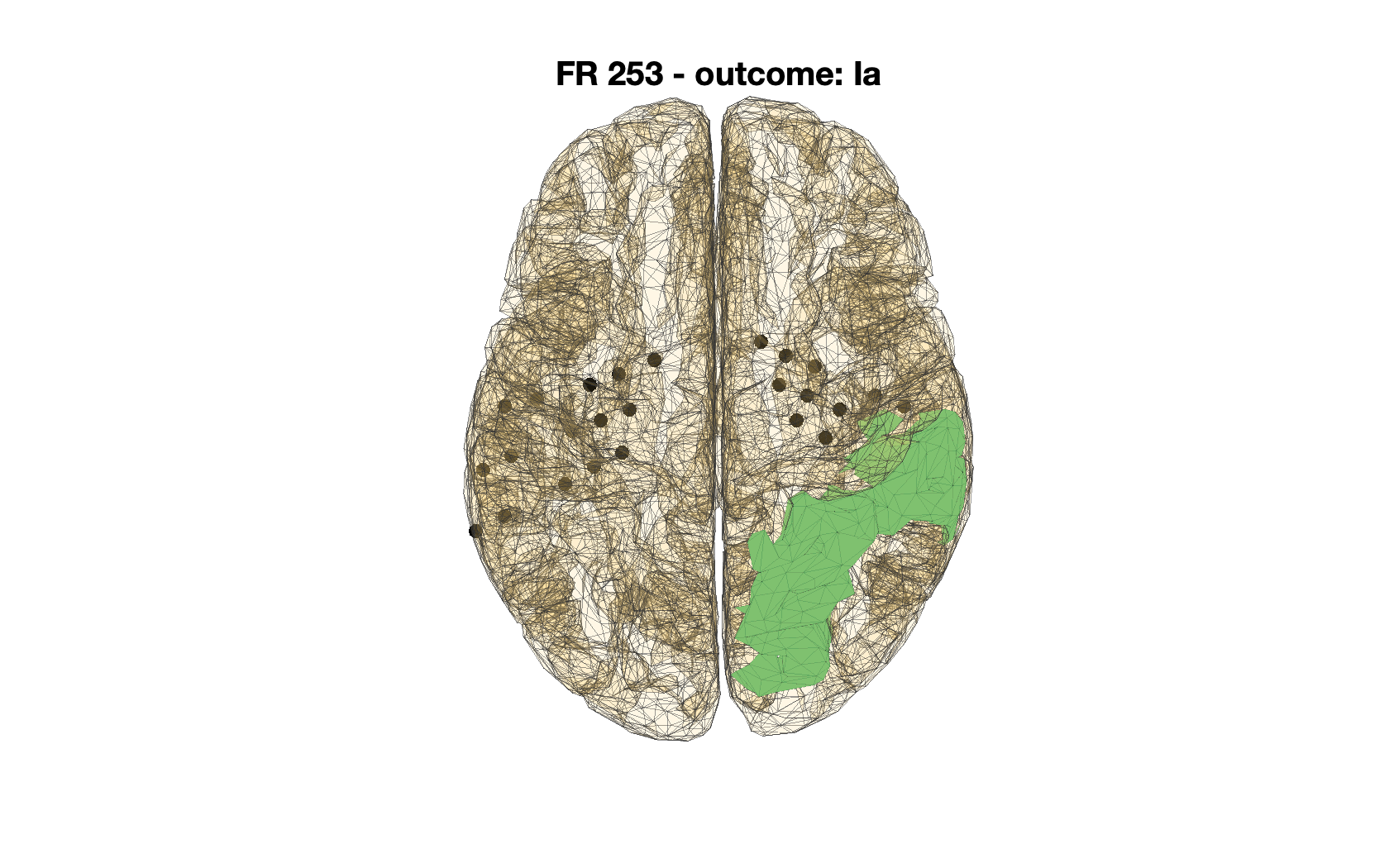

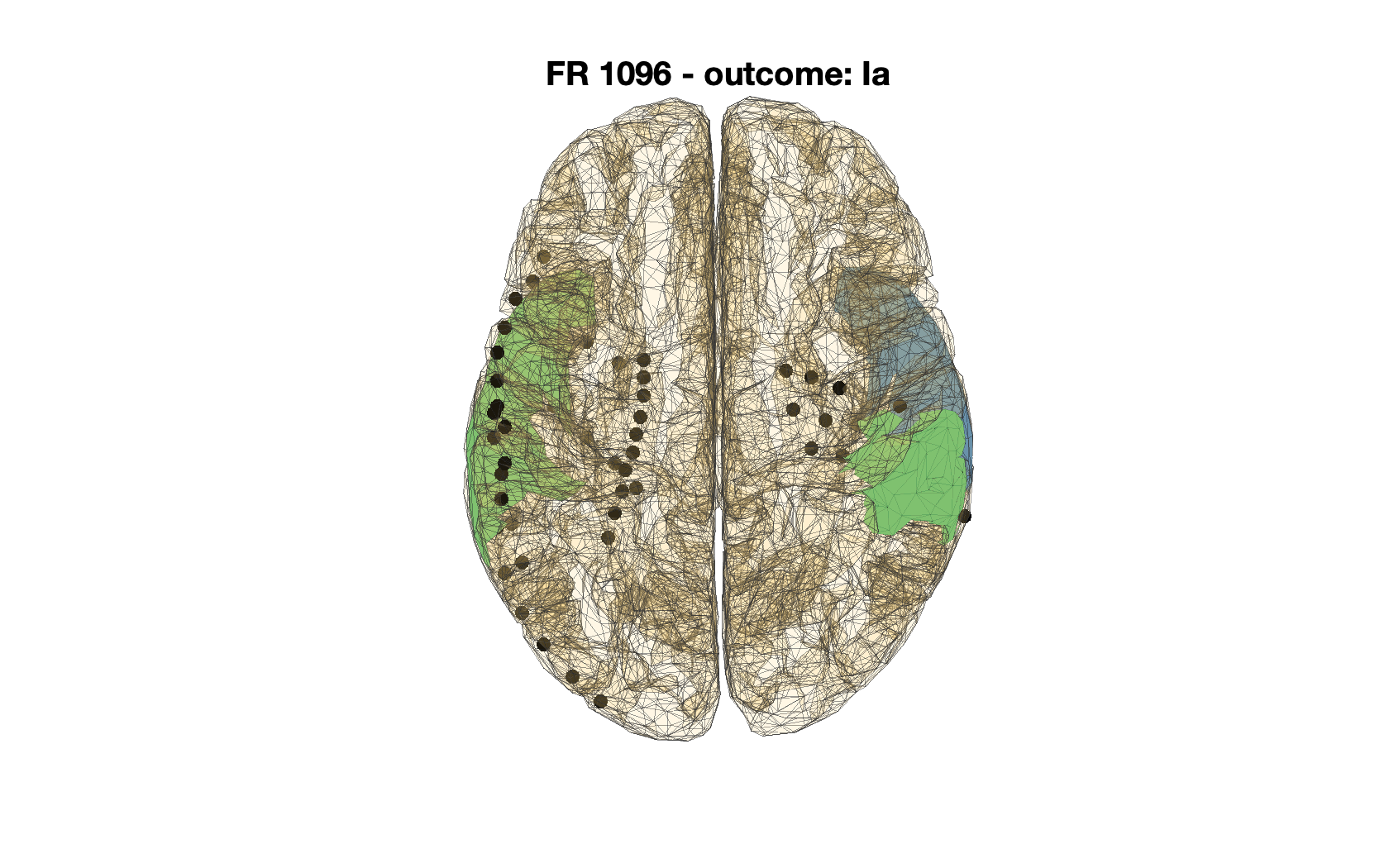

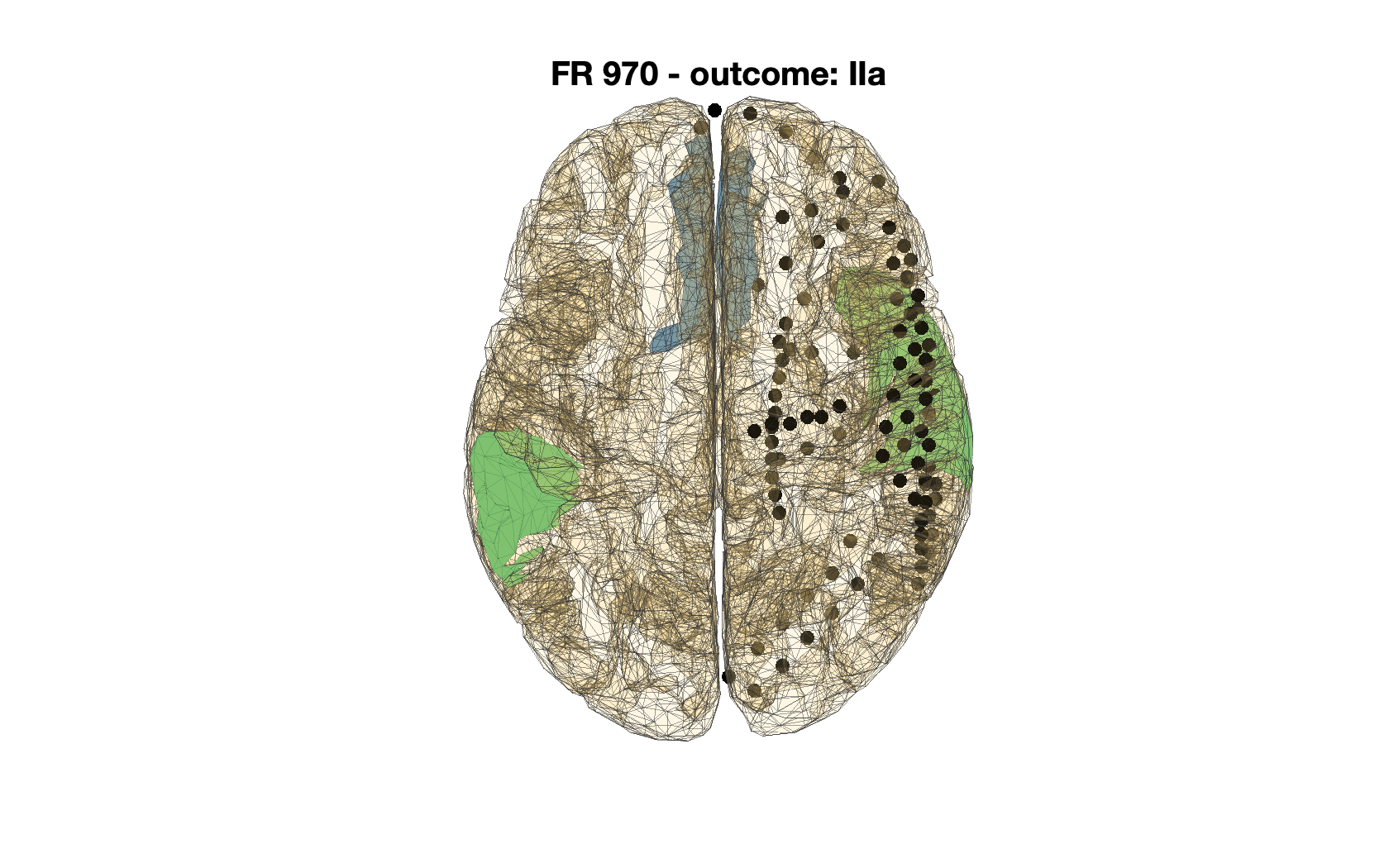

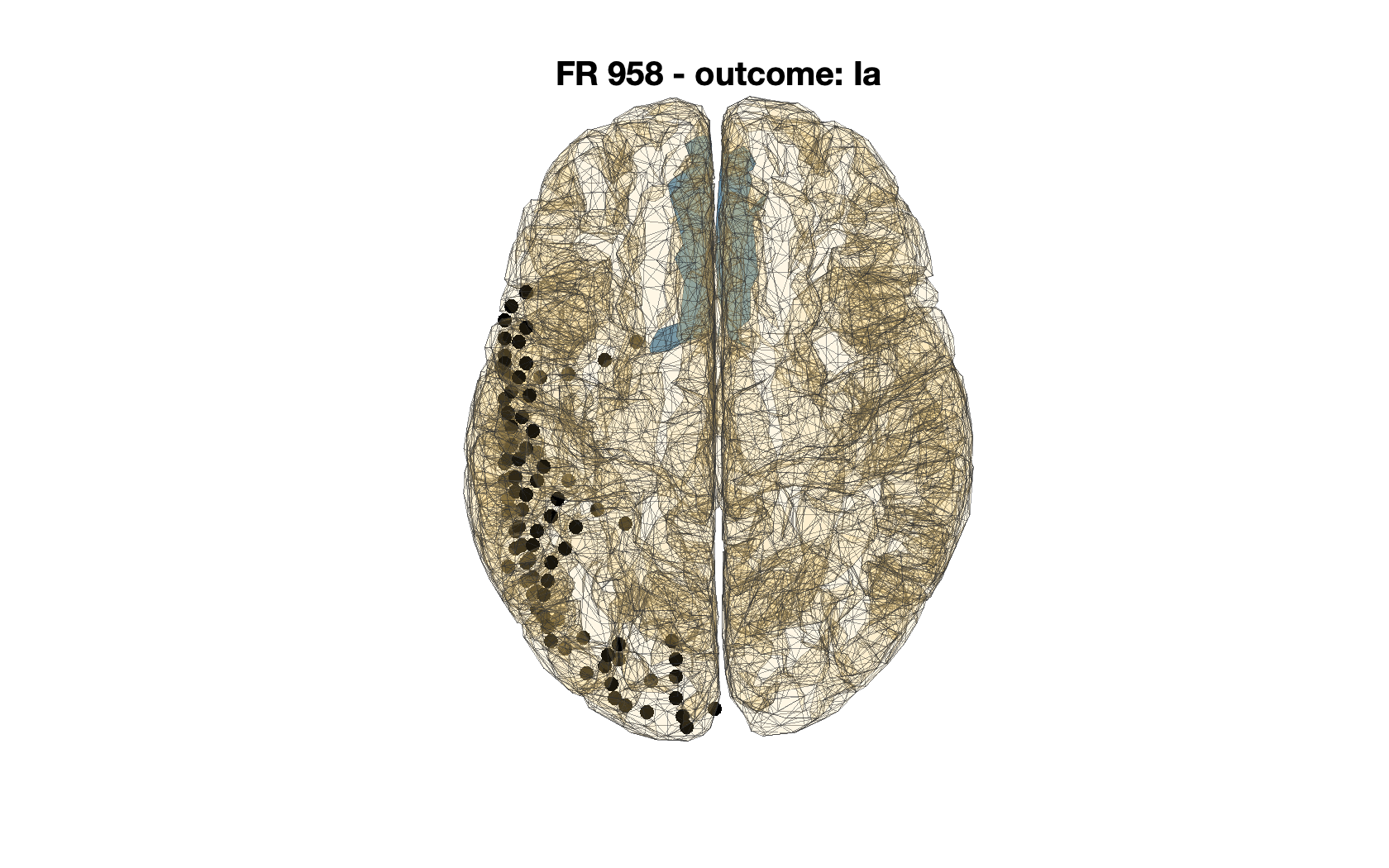

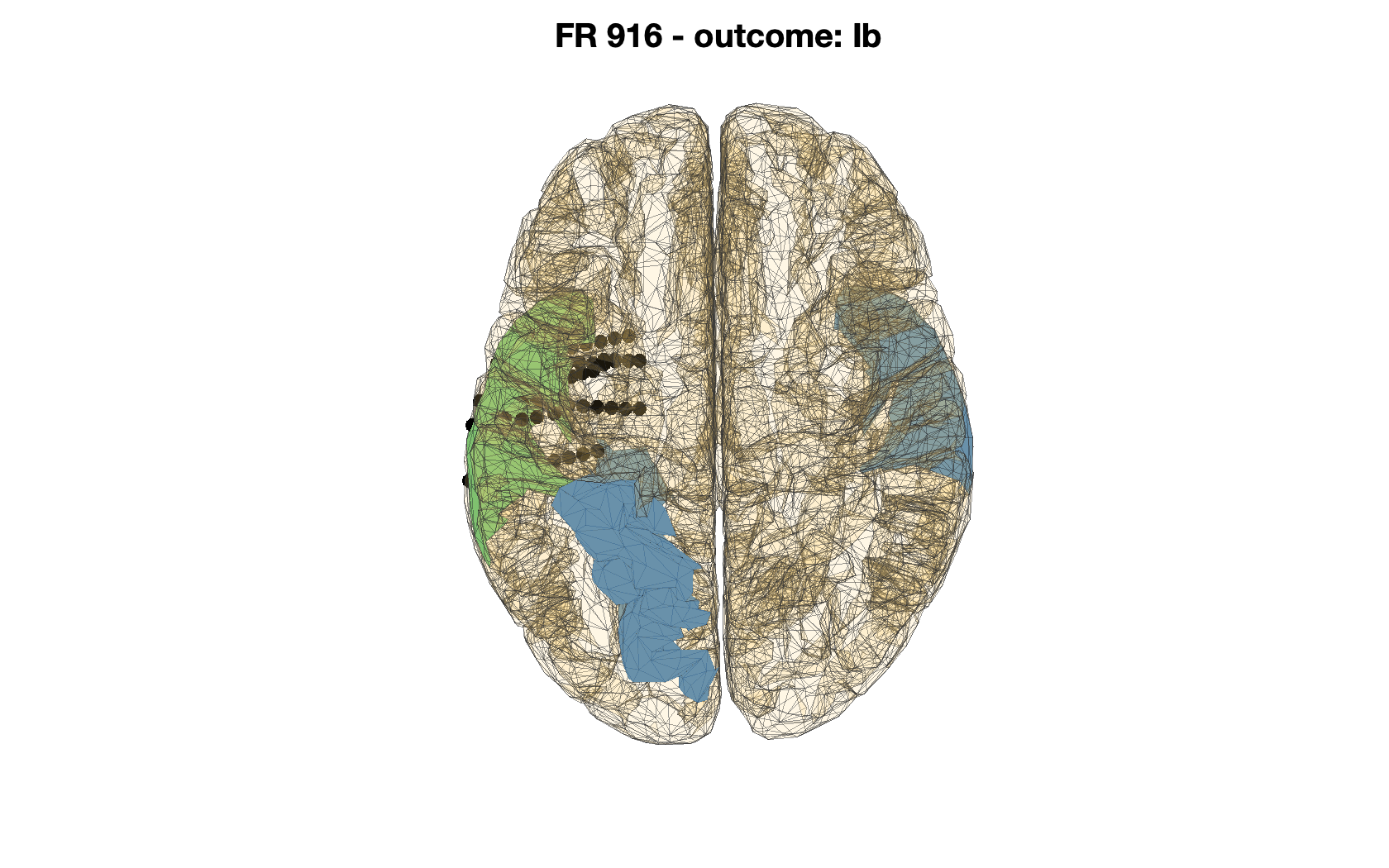

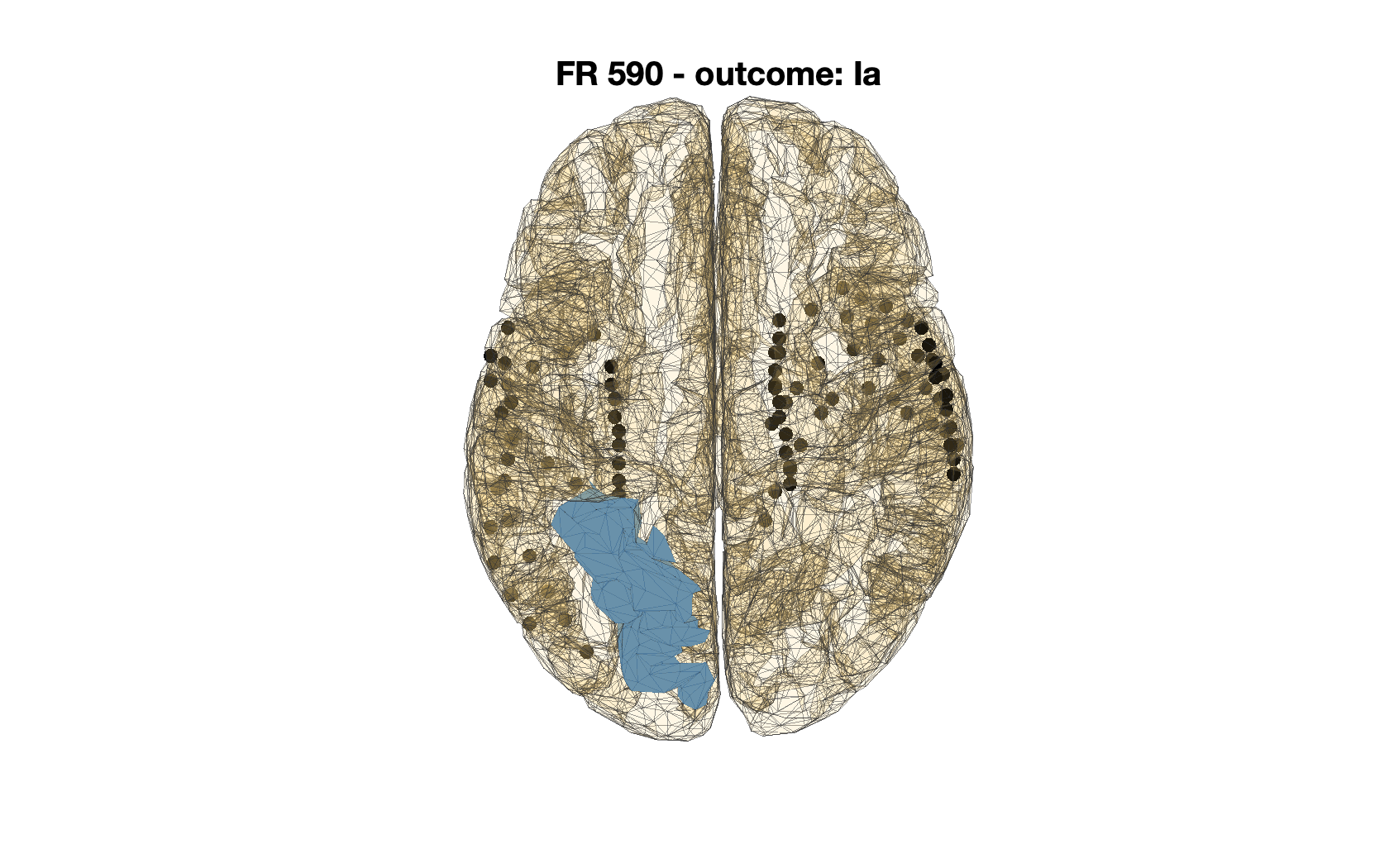


**Figure S1**

These figures are equivalent to the two panels in Figure 2 and correspond to all individuals considered in this study. As described in the caption of Figure 2, the black dots represent electrodes. ROIs highlighted in blue were identified by the framework from one seizure epoch, whereas ROIs highlighted in green were identified from two different seizure epochs. The highlighted regions are named in Supplementary Table 1.
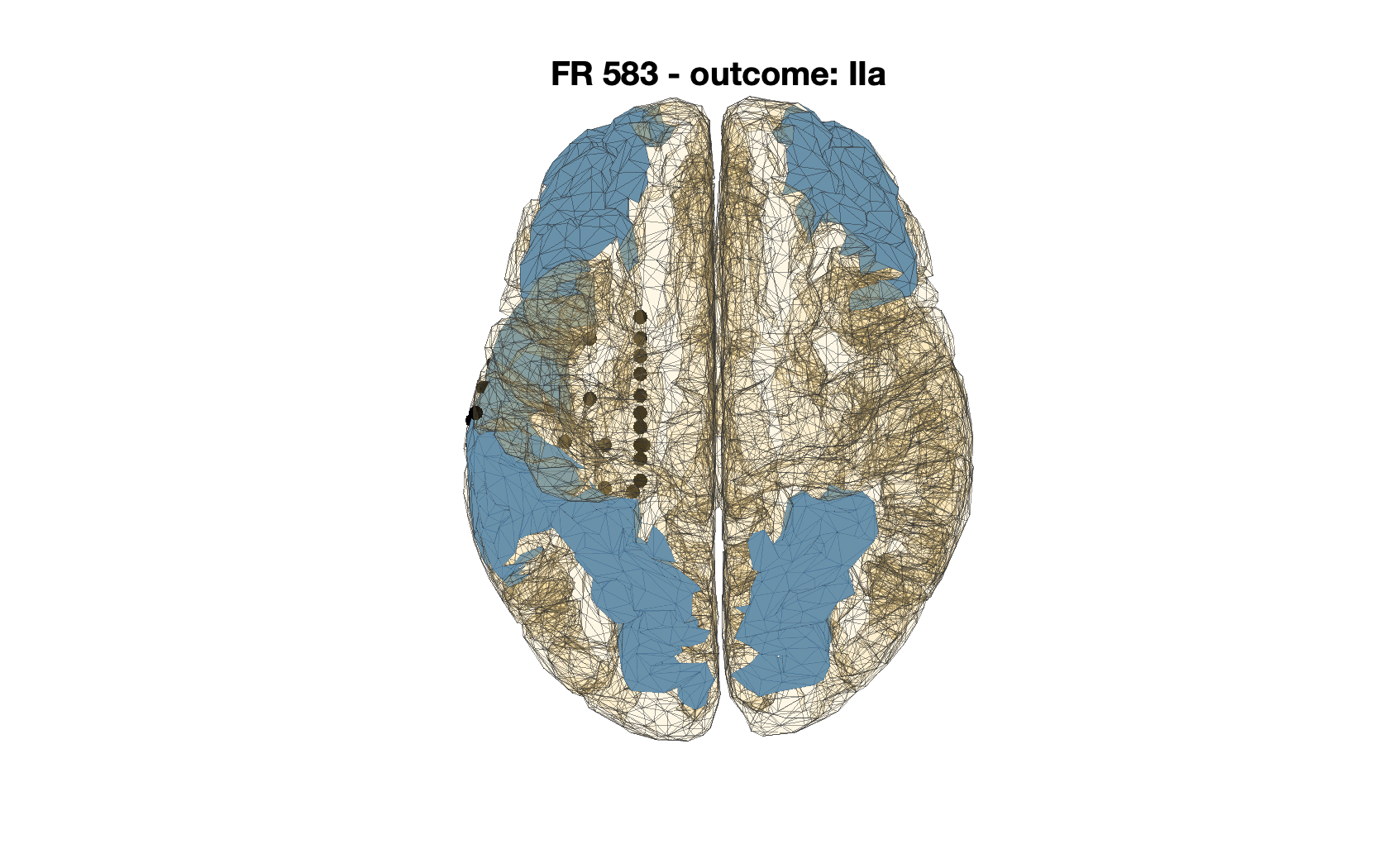

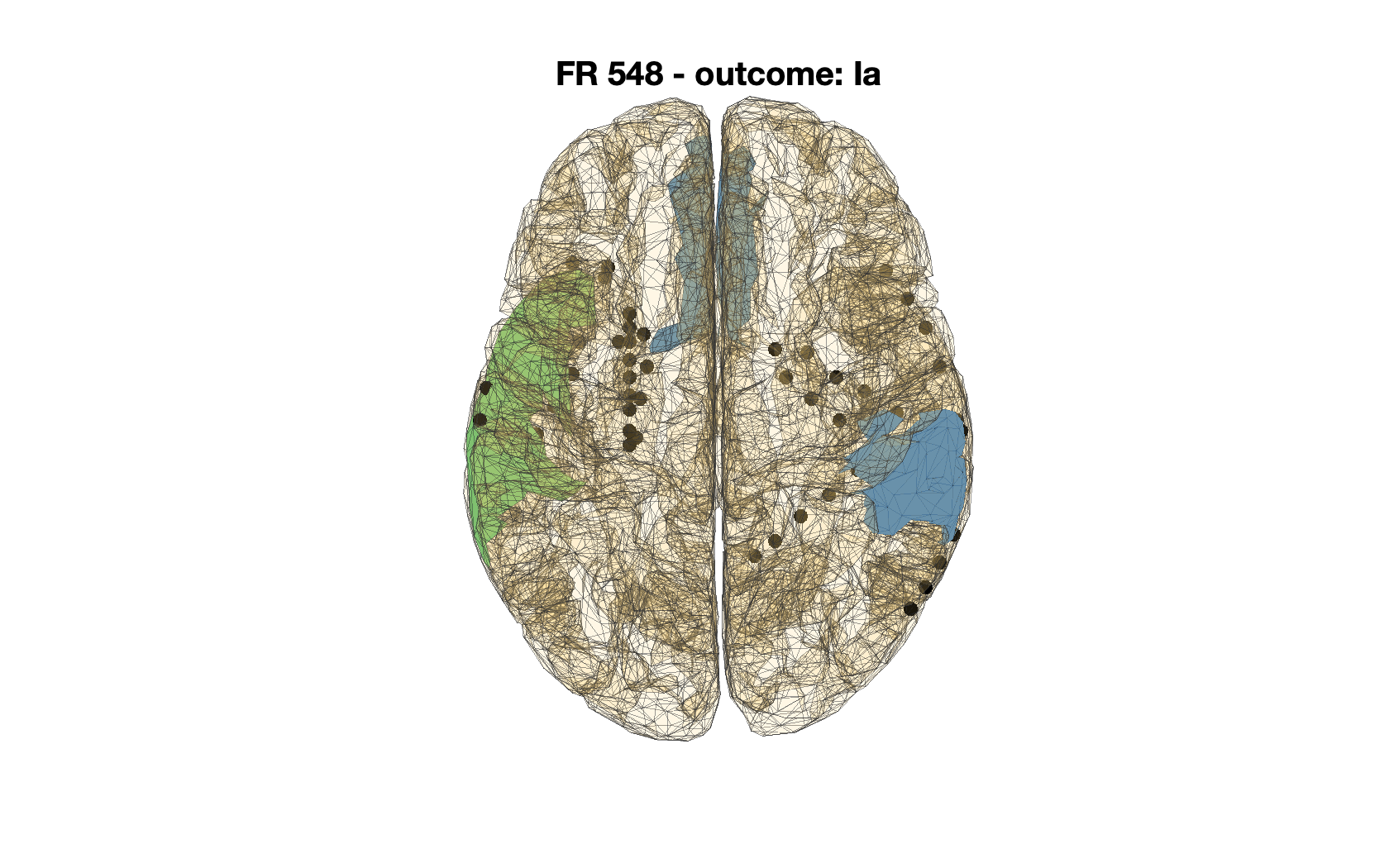

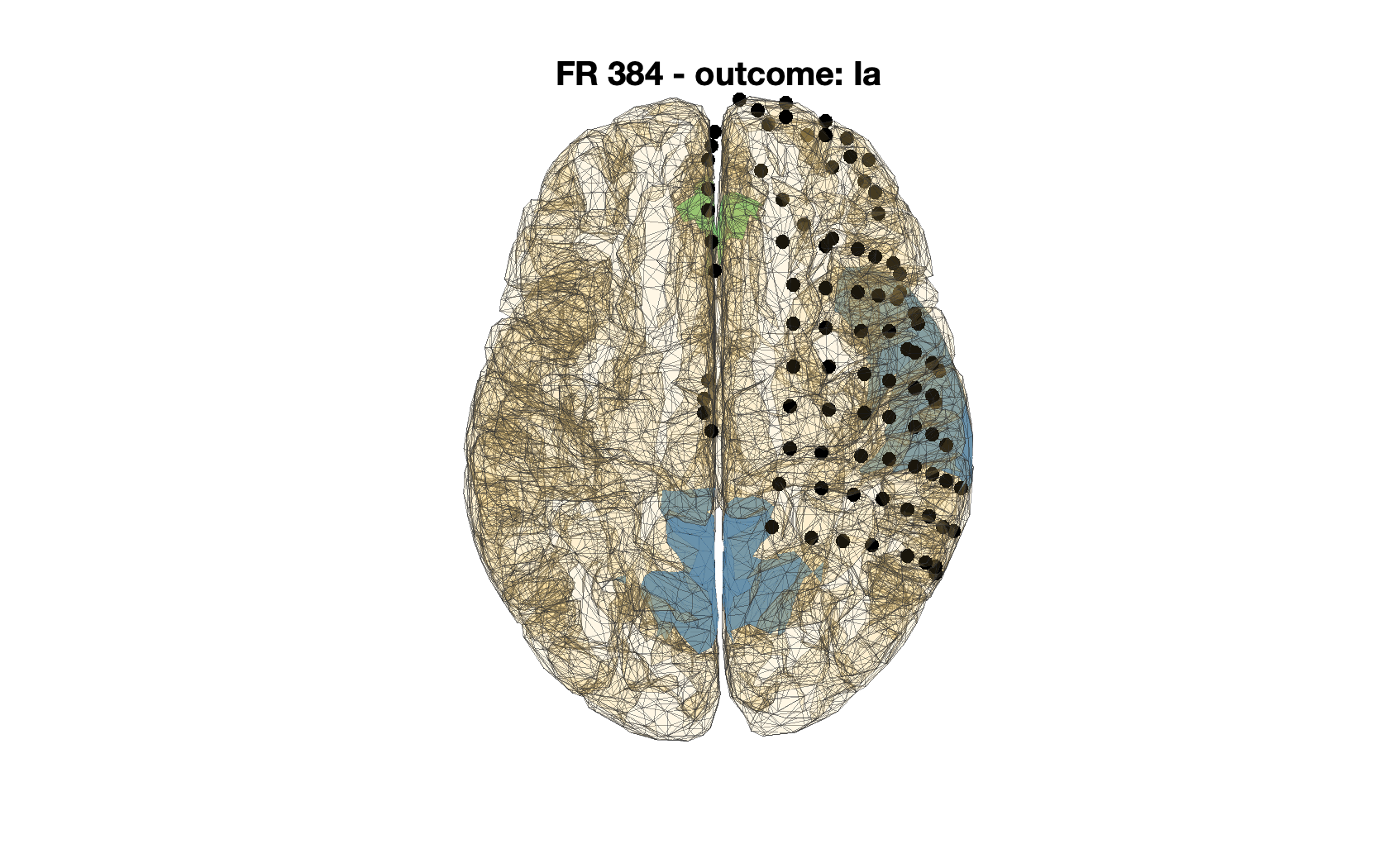

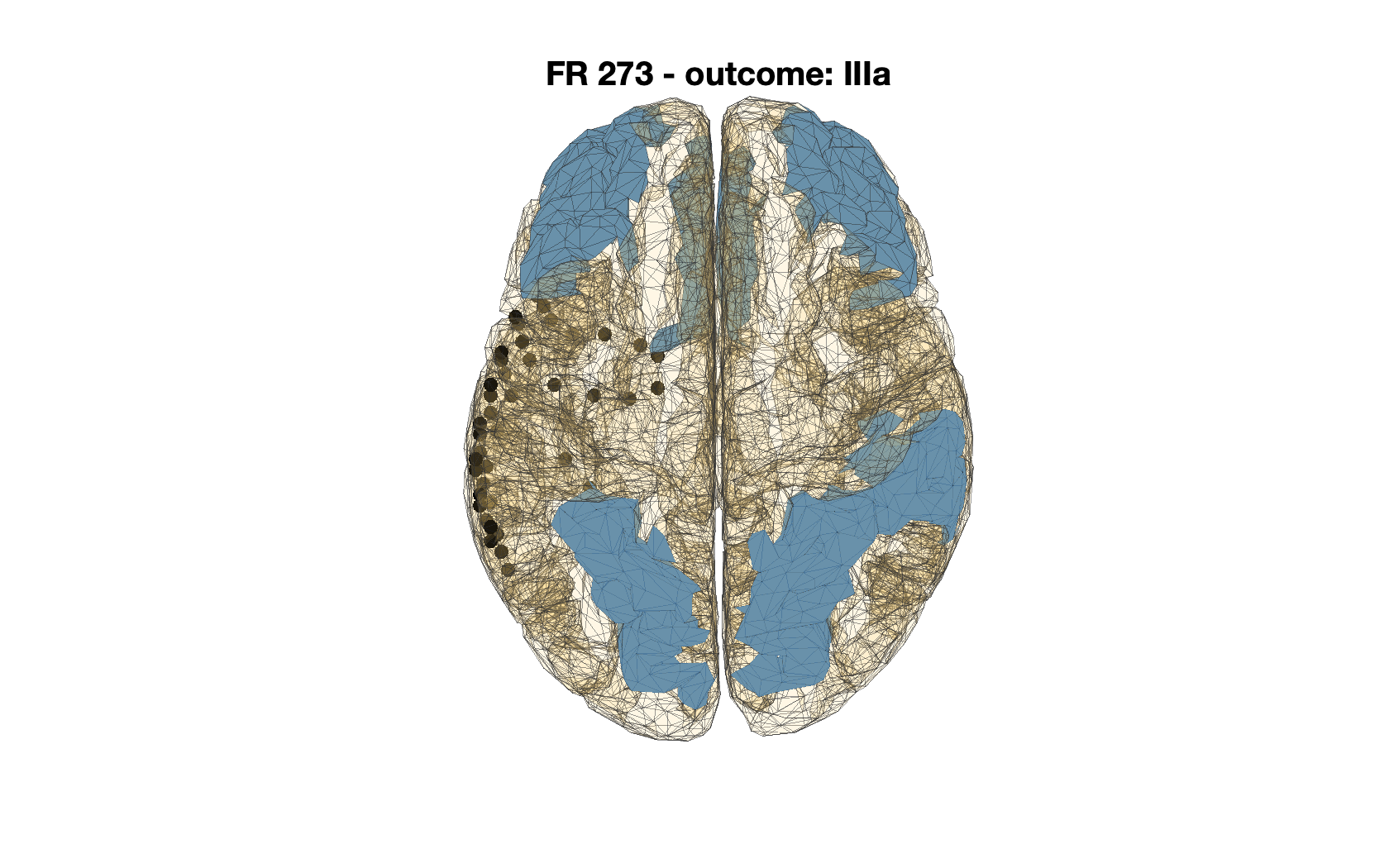

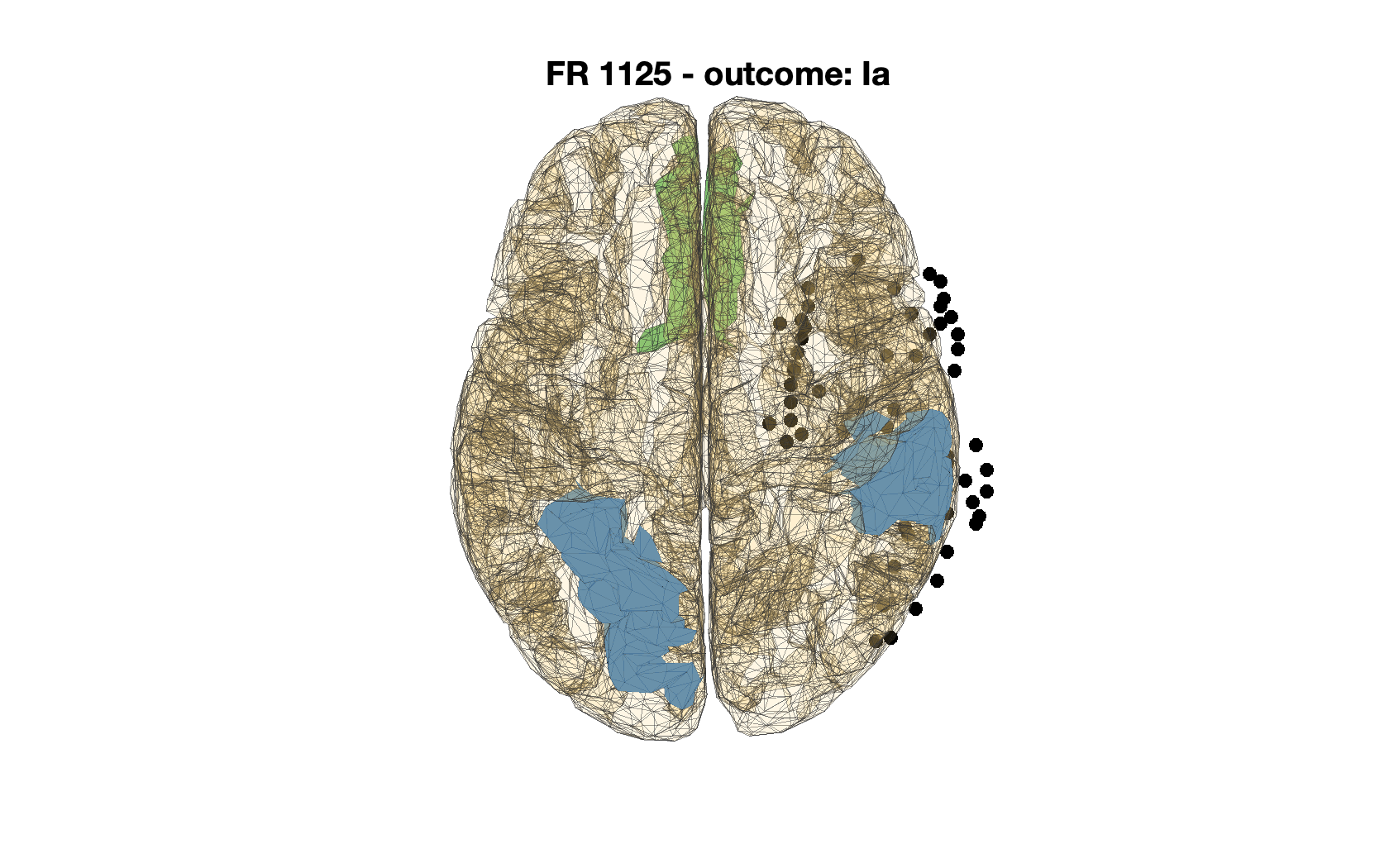

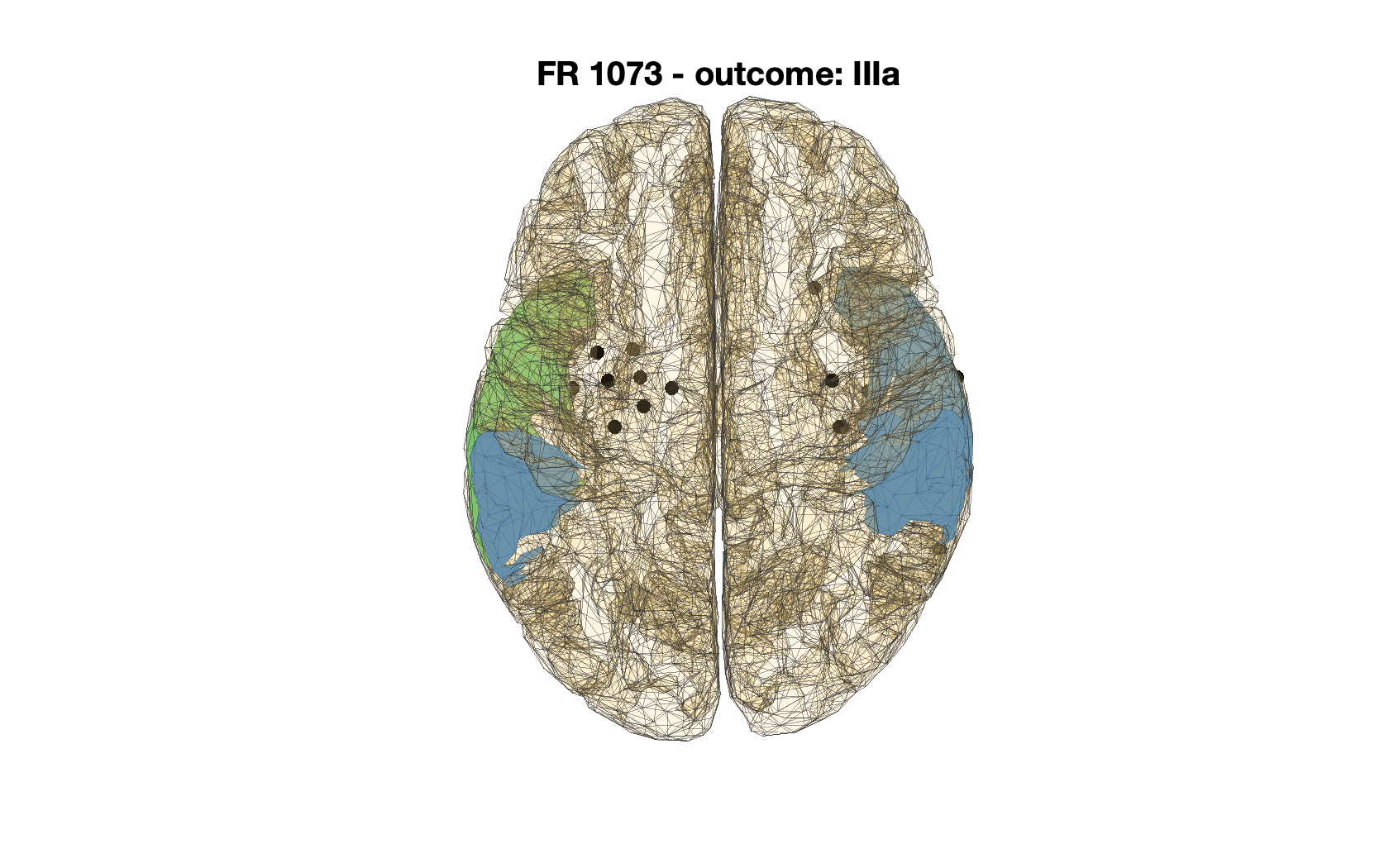


**Figure S1 (cont.)**


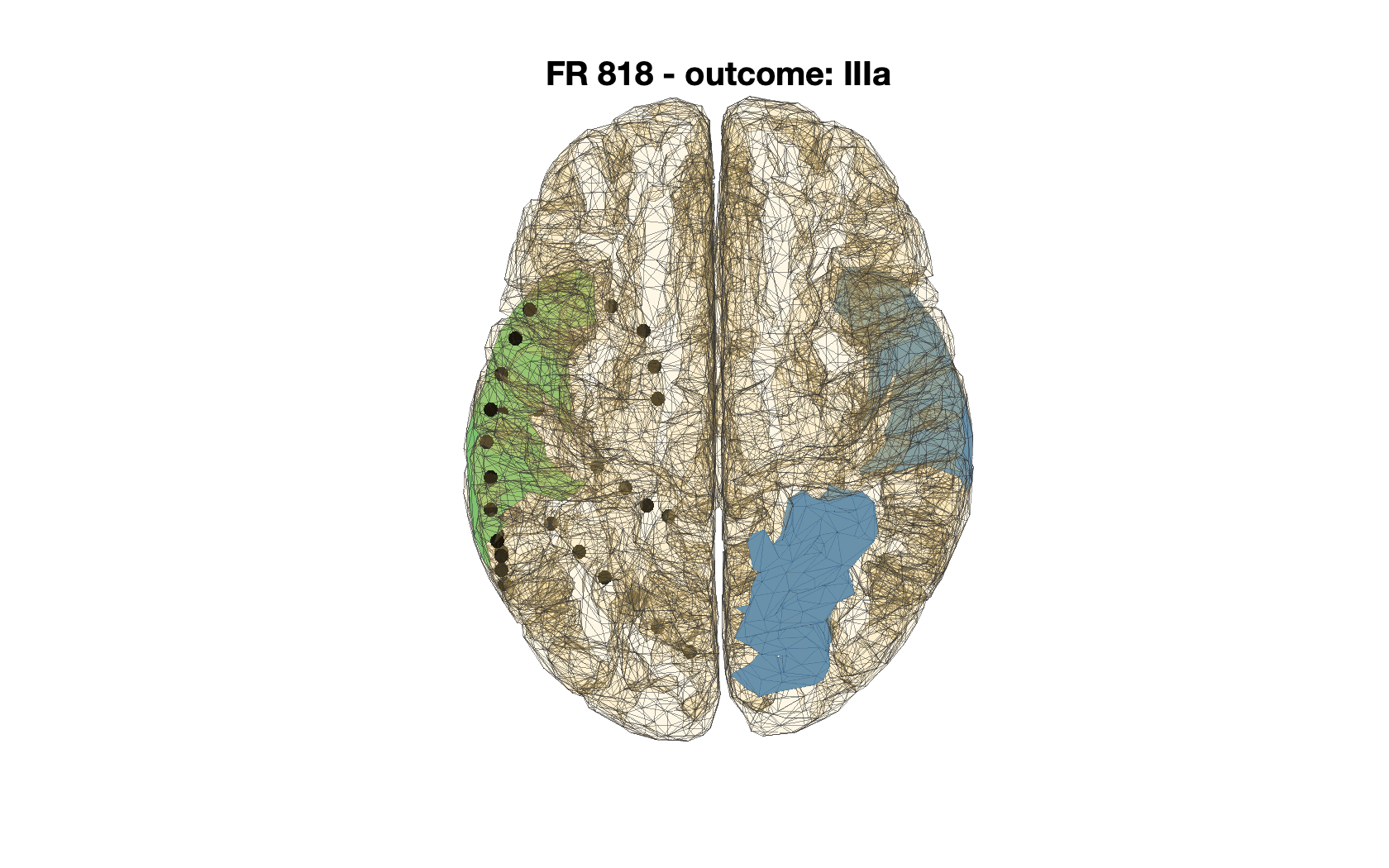

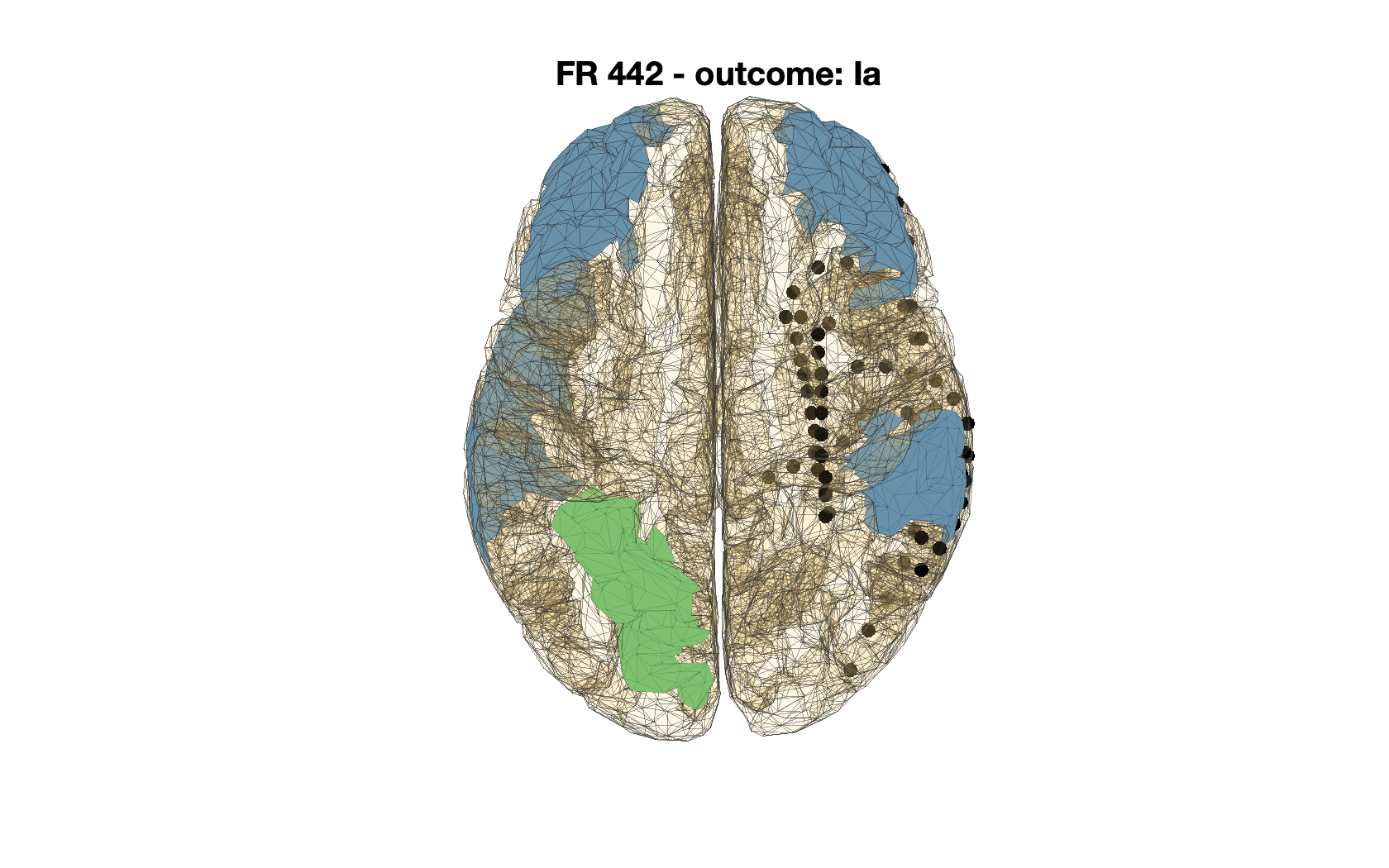

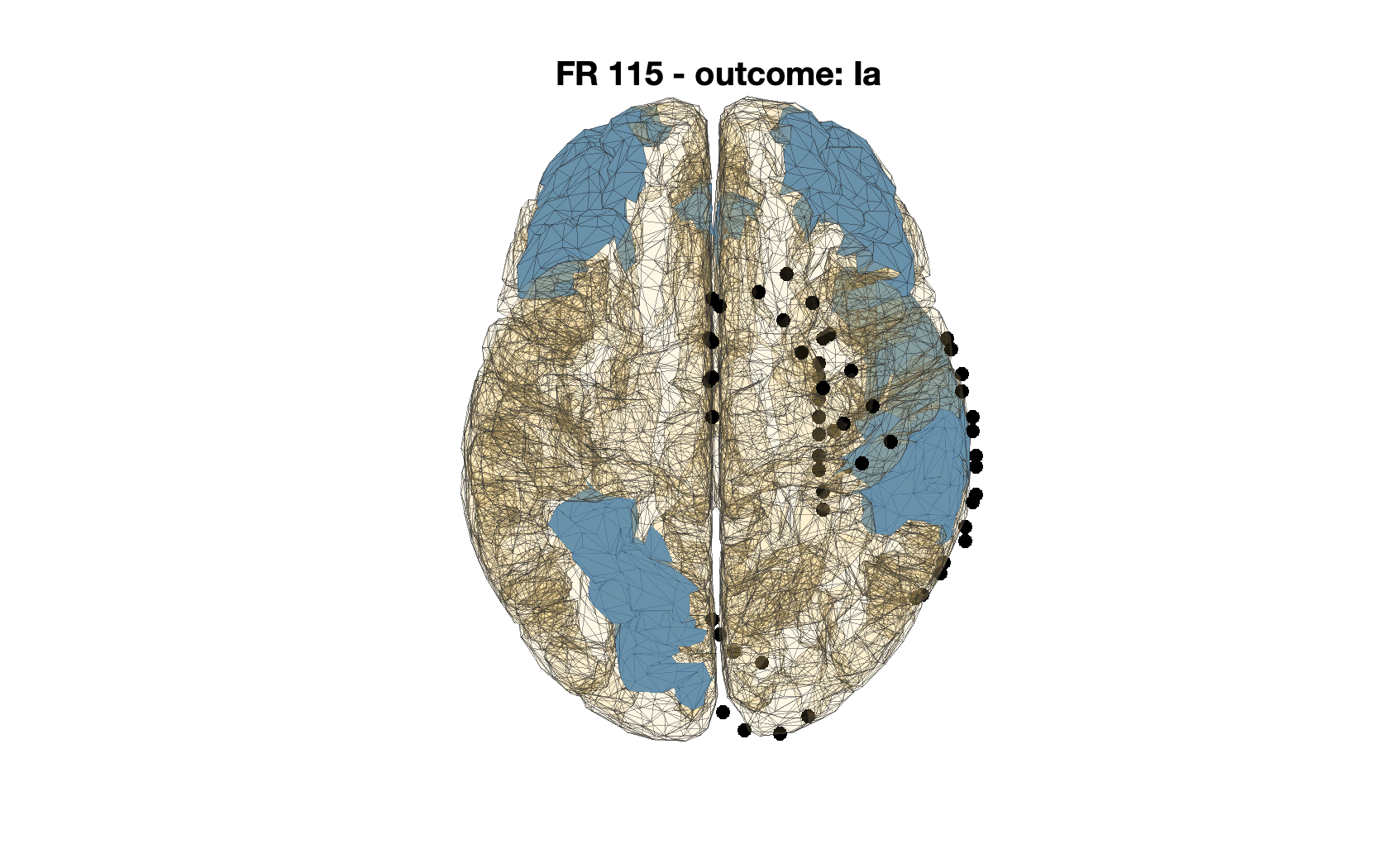


**Figure S1 (cont.)**
