# Supplementary material for: Computational modelling in source space from scalp EEG to inform presurgical evaluation of epilepsy
Source: Clin Neurophysiol. 2020 Jan;131(1):225–34. doi: 10.1016/j.clinph.2019.10.027 (PMC6941468; doi:10.1016/j.clinph.2019.10.027)
Supplement: Supplementary data 2 [file mmc2.docx]

| Patient ID | Outcome | Electrode implantation | Surgery localization | ROIs with highest NI |
| --- | --- | --- | --- | --- |
| FR 115 | Ia | right | temporal right | superior parietal left  supramarginal right  superior temporal right  rostral anterior cingulate  rostral middle frontal |
| FR 253 | Ia | bilateral | temporal right | superior parietal right  supramarginal right  supramarginal right  superior parietal right |
| FR 384 | Ia | right | frontal right | precuneus  rostral anterior cingulate  rostral anterior cingulate  superior temporal right |
| FR 442 | Ia | right | temporal right | superior temporal left  rostral middle frontal  supramarginal right  superior parietal left  superior parietal left |
| FR 548 | Ia | bilateral | temporal left | superior temporal left  medial orbitofrontal  superior temporal left  supramarginal right |
| FR 590 | Ia | bilateral | temporal left | superior parietal left |
| FR 916 | Ib | left | temporal left | superior parietal left  superior temporal left  superior temporal left  parahippocampal left  superior temporal right |
| FR 958 | Ia | left | none (no MRI) | medial orbitofrontal |
| FR 1096 | Ia | bilateral | temporal left | superior temporal left  superior temporal left  supramarginal right  supramarginal right  superior temporal right |
| FR 1125 | Ia | right | temporal right | medial orbitofrontal  medial orbitofrontal  supramarginal right  superior parietal left |
| FR 273 | IIIa | left | temporal left | superior parietal left  superior parietal right  medial orbitofrontal  rostral middle frontal  supramarginal right |
| FR 583 | IIa | left | temporal left | supramarginal left  rostral middle frontal  superior parietal left  superior parietal right  superior temporal left |
| FR 818 | IIIa | left | temporal left | superior temporal left  superior temporal right  superior temporal left  superior parietal right |
| FR 970 | IIa | right | temporal right | medial orbitofrontal  supramarginal left  superior temporal right  superior temporal right  supramarginal left |
| FR 1073 | IIIa | bilateral | temporal right | superior temporal left  supramarginal right  superior temporal right  supramarginal left  superior temporal left |

**Table S1**

Clinical characteristics of the individuals considered in this study and ROIs with highest NI per seizure epoch. As in Table 1 and 3, the first column identifies the patients’ ID. The outcome column describes their postsurgical outcome. The electrode implantation column specifies whether intracranial electrodes were implanted either in the right or in the left hemispheres or both (bilateral). Surgery localisation defines the brain region targeted by the performed surgery (established from an MRI after surgery). The last column indicates the ROIs with highest NI in each seizure considered per individual.
